# Back to translation: removal of aIF2 from the 5′-end of mRNAs by translation recovery factor in the crenarchaeon *Sulfolobus solfataricus*

**DOI:** 10.1093/nar/gkt1169

**Published:** 2013-11-23

**Authors:** Birgit Märtens, Salim Manoharadas, David Hasenöhrl, Lukas Zeichen, Udo Bläsi

**Affiliations:** Department of Microbiology, Immunobiology and Genetics, Max F. Perutz Laboratories, Center of Molecular Biology, University of Vienna, Dr. Bohrgasse 9, 1030 Vienna, Austria

## Abstract

The translation initiation factor aIF2 of the crenarchaeon *Sulfolobus solfataricus* (Sso) recruits initiator tRNA to the ribosome and stabilizes mRNAs by binding via the γ-subunit to their 5′-triphosphate end. It has been hypothesized that the latter occurs predominantly during unfavorable growth conditions, and that aIF2 or aIF2-γ is released on relief of nutrient stress to enable in particular anew translation of leaderless mRNAs. As leaderless mRNAs are prevalent in Sso and aIF2-γ bound to the 5′-end of a leaderless RNA inhibited ribosome binding *in vitro*, we aimed at elucidating the mechanism underlying aIF2/aIF2-γ recycling from mRNAs. We have identified a protein termed Trf (translation recovery factor) that co-purified with trimeric aIF2 during outgrowth of cells from prolonged stationary phase. Subsequent *in vitro* studies revealed that Trf triggers the release of trimeric aIF2 from RNA, and that Trf directly interacts with the aIF2-γ subunit. The importance of Trf is further underscored by an impaired protein synthesis during outgrowth from stationary phase in a Sso *trf* deletion mutant.

## INTRODUCTION

In the model crenarchaeon *Sulfolobus solfataricus* (Sso), two different modes of translation initiation have been described (1,2). One is based on a Shine-Dalgarno/anti-Shine-Dalgarno interaction and operates at internal cistrons of polycistronic mRNAs. In contrast, monocistronic mRNAs and proximal genes of polycistronic mRNAs are frequently devoid of a 5′-untranslated region (3). In fact, in the majority of the protein-coding transcripts (69%), the transcriptional start site was mapped to the adenosine of the predicted start codon or 1–3 bases upstream (3). Decoding of these leaderless mRNAs appears to depend—analogously to Bacteria (4)—on base-pairing of the start codon with the anti-codon of initiator tRNA (tRNAi) residing in the P-site of the 70S ribosome (2).

Translation initiation in Sso depends on several translation initiation factors (IFs), some of which have eukaryal but no bacterial counterparts (1,5). Both Archaea and Eukaryotes use the heterotrimeric translation initiation factor 2 (e/aIF2) to recruit tRNAi to the translation initiation complex (6–8). In Eukaryotes, the trimeric structure of eIF2 is of functional importance. The eIF2-γ subunit contains the GTP/GDP binding site and participates in tRNAi binding (6,8). The eIF2-β subunit contributes to tRNAi binding and includes several domains important for the interaction with other factors, such as the guanine nucleotide exchange factor eIF2B and the GTPase activator eIF5 (6,8). The eIF2-α subunit contains a phosphorylation site. Phosphorylation of Ser51 prevents the exchange of GDP with GTP, and eIF2 remains locked in the inactive GDP-bound form, which in turn impedes translation initiation, e.g. during nutrient stress (6,8).

Like eukaryotic eIF2, Sso aIF2 consists of three polypeptides. In contrast to eIF2 (6,8), aIF2 does not serve as a shuttle for tRNAi, but ribosome-bound aIF2 recruits tRNAi to the ribosome, which complies with the mode of tRNAi recruitment in bacteria (9,10). In Sso aIF2, the α- and γ-subunits interact with tRNAi (11,12), and were shown to be sufficient for tRNAi binding (7). Phosphorylation of the α-subunit has been reported for aIF2 of *Pyrococcus horikoshii* (13) and that of *Thermococcus kodakaraensis* (14) but not for aIF2-α of Sso. The archaeal aIF2-β subunit lacks the N-terminal domain of eIF2-β, which is required for the interaction with eIF2B and eIF5 (8). However, the Zn-binding domain, assisting eIF2 in tRNAi binding is present in aIF2-β, albeit Sso aIF2-β is not required for tRNA binding (7).

Sso aIF2 is a moonlighting protein. In addition to recruiting tRNAi on the ribosome (9), aIF2 was shown to bind via its γ-subunit to the 5′-triphosphate end (5′-P_3_-end) of mRNAs and to protect RNAs from 5′ to 3′ directional decay by the exoribonuclease aCPSF2 (formerly termed Sso RNase J) (15,16). The affinity of both, trimeric aIF2 and aIF2-γ, is higher for the 5′-P_3_-end of RNA than for tRNAi, whereas in the presence of aIF1 and aIF1A ribosome bound trimeric aIF2 has a higher affinity for tRNAi than for the 5′-P_3_-end of RNA (9). Thus, we hypothesized that aIF2 fulfils its task as a translation IF as long as protein synthesis prevails, whereas during nutrient starvation, i.e. when translation is limited, aIF2 and/or aIF2-γ alone (aIF2/aIF2-γ) bind to the 5′-P_3_-end of mRNAs (9,15). Moreover, the model specified that aIF2/aIF2-γ is released on relief of nutrient stress to enable in particular anew translation of leaderless mRNAs (15).

In this study we addressed the question whether aIF2/aIF2-γ release from the 5′-P_3_-end of RNA is mediated by modifications of the aIF2 γ-subunit or by a proteinaceous factor. We show that Sso protein 2509 is associated with aIF2 during outgrowth from prolonged stationary phase, and that Sso2509 and aIF2-γ interact directly. Moreover, *in vitro* studies revealed that Sso2509 triggers aIF2 release from the 5′-P_3_-end of RNAs, and deletion of Sso ORF *2509* impaired global protein synthesis during outgrowth. As protein Sso2509 allowed for a faster resumption of protein synthesis after stress relief, the protein was termed Trf (translation recovery factor).

## MATERIALS AND METHODS

### Strains and plasmids

All Sso strains used in this study are listed in Supplementary Table S1. If not indicated otherwise, the cells were grown in Brock’s medium (composed of Brock’s salts and supplemented with 0.2% NZamine and 0.2% sucrose). The Sso *2509* deletion strain PBL2025Δ *2509* and plasmid pMJ05- γ_His_ were constructed as described in Supplementary Materials.

### Purification of protein 2509, Sso-aCPSF2 and aIF2

Sso ORF *2509* was amplified by polymerase chain reaction (PCR) using genomic DNA of Sso strain P2 and the oligonucleotides 5′-CATGCCATGGATATGAGGGAAGAAGAAATAAG-3′ (contains a NcoI restriction site) and 5′-GGACTAGTTTAAAAGTTATGCAAATGTG-3′ (contains a SpeI restriction site). The PCR product was cleaved with NcoI and SpeI, and ligated into the corresponding sites of plasmid pPROEX HTb (Invitrogen), resulting in plasmid pPROEX-2509_His_.

Recombinant Sso protein 2509 was purified from *Escherichia coli* by Ni-NTA affinity chromatography (Qiagen) following standard protocols using lysis buffer A (50 mM Tris–HCl, pH 8.0, 300 mM NaCl, 15 mM imidazole, 10 mM β-mercaptoethanol), wash buffer B (50 mM Tris–HCl, pH 8.0, 1 M NaCl, 15 mM imidazole, 10 mM β-mercaptoethanol) and elution buffer (50 mM Tris–HCl, pH 8.0, 300 mM NaCl, 250 mM imidazole). The protein was dialyzed against buffer (50 mM Tris, pH 6.0, 100 mM KCl, 5% glycerol; 1 mM MgCl_2_; 10 mM imidazole, 1 mM DTT) and stored at −20°C.

Sso-aCPSF2 and the aIF2 subunits were purified as previously reported (15,16).

### Toeprinting

The PCR-template for *in vitro* synthesis of leaderless *104* mRNA was prepared using plasmid pBS800 (17) as template together with the oligonucleotides 5′-AGATAATACGACTCACTATAGATGTCTCAAAGTTTTGAGGGAGAAT-3′ containing a T7 Promotor (underlined) and the reverse primer 5′-GCACTCATTGCTTCACCTCTTTAAT-3′. The RNA was synthesized *in vitro* by using the PCR template and T7 RNA polymerase (Fermentas), and then gel purified. The leaderless mRNA designated *104* (0.05 nM) (encoding ribosomal protein L30e) was annealed with [^32^P]-5′-end-labeled oligonucleotide 5′-GTTCCTAAAATTACTTTGCC-3′ complementary to nt +46—+65 downstream of the A (+1) of the start codon of RNA *104*. The annealing mix was then incubated for 10 min with and without 1 µM aIF2-γ at 65°C. Primer extension was performed with reverse transcriptase M-MLV (Promega) at 37°C in 10 mM Tris, pH 7.5, 60 mM NH_4_Cl, 6 mM β-mercaptoethanol, 10 mM MgOAc. Toeprinting (18) was performed in the presence of *E. coli* 70 S monosomes (0.4 µM) and tRNA_f-Met_ (1.6 µM) under the same conditions. The resulting cDNA sequence 5′-gATGTCTCAAAGTTTTGAGGGAGAATTAAAAACACTTCTTAGAAGTGGCAAAGTAATTTTAGGAAC-3′ obtained after primer extension comprises 65 nt of the 5′ part of *104* RNA plus a G at position −1 as a result from *in vitro* transcription.

### Capturing of Sso2509 (Trf) by immobilized aIF2

The Sso strains PH1-16(pMJ05) (19) and PH1-16(pMJ05-γ_His_) were grown in Brock’s medium to an OD_600_ of 0.6. The cells were harvested and reinoculated in Brock’s medium containing 0.2% NZamine without sucrose. After the cells reached an OD_600_ of 0.5, the cells were again harvested and reinoculated in 300 ml of Brock’s medium supplemented with 0.2% NZamine and 0.2% arabinose. After growth of strain PH1-16(pMJ05-γ_His_), to stationary phase (OD_600_ = 1.5) and further incubation in the same medium for 3 days (prolonged stationary phase), 50 ml of culture were withdrawn and used to isolate His-tagged aIF2 by means of Ni-affinity chromatography. In addition, 50 ml of culture from the same strain were harvested and then reinoculated in 300 ml of arabinose-containing medium. His-tagged aIF2 was then isolated by means Ni-affinity chromatography from 300 ml of culture of strain PH1-16(pMJ05-γ_His_) 3 h after dilution into fresh medium (outgrowth). Furthermore, a mock purification was performed with 300 ml of culture of strain PH1-16(pMJ05), which was cultivated in the same manner and subjected to outgrowth as the latter culture. The cell pellets were lysed using buffer A (50 mM Tris–HCl, pH 8.0, 300 mM NaCl, 15 mM imidazole, 10 mM β-mercaptoethanol). Buffer A was used as washing buffer, and buffer A containing 250 mM imidazol was used for elution. The eluate was TCA precipitated and separated using a 12% Tris/Tricine gel, which was stained with Coomassie brilliant blue. The band corresponding to protein Sso2509 was excised and subjected to mass spectrometry.

### RNA-biotinylation and aIF2 release by Sso2509

The 2508sh RNA was prepared as described before (15). Hundred picomoles 2508sh RNA and 300 pmol biotinylated oligonucleotide 5′-CAGGTGGCACTTTTCGGG(biotin)-3′ were ligated, using T4-RNA-ligase (Thermo scientific). The ligation mix was separated on a 6% PAA/8 M urea gel, and the biotinylated 2508sh mRNA was gel purified. The biotinylated 2508sh RNA was bound to 100 µl of Dynabeads® Straptavidin beads (Invitrogen) according to the manufacturer’s instructions. The RNA-coated beads were equilibrated in Co-IP (co-immunoprecipitation) buffer (50 mM Tris, pH 6.0, 100 mM KCl, 5% glycerol; 1 mM MgCl_2_). Hundred picomoles aIF2-α, -β, -γ were incubated for 10 min at 65°C to allow trimer formation, cooled to 4°C and then added to the RNA-coated beads and incubated for 2 h at 4°C. The unbound protein(s) in the supernatant (S) were collected and the beads were washed six times with 1 ml of IP-buffer. TCA (10%) was added to the last wash fraction (W) to ensure complete removal of aIF2 as judged by western blot analysis. The beads were resuspended in 200 µl of either IP buffer alone or in the presence of protein Sso2509 (100 pmol), and incubated for 2 h at 4°C. After incubation, the released fraction (Figure 2B: S) was collected, the beads were washed three times (Figure 2B: W) and the beads were boiled in sodium dodecyl sulphate (SDS)-loading buffer (Figure 2B: B). The supernatants and wash fractions were precipitated with 10% TCA and boiled in loading buffer. The samples were separated on a 12% SDS-polyacrylamide gel and then transferred to a nitrocellulose membrane by electroblotting. The aIF2 subunits were detected using aIF2-α, -β and -γ-specific antibodies and an anti-rabbit antibody coupled to alkaline phosphatase followed by visualization of the immunocomplexes with the substrate NBT/BCIP using standard procedures.

### Co-immunoprecipitation assay

Hundred picomoles of trimeric aIF2, aIF2-α, -β and -γ subunit, respectively, were incubated alone (control) or together with 100 pmol Sso2509 in 200 µl of co-immunoprecipitation (Co-IP) buffer (50 mM Tris, pH 6.0, 100 mM KCl, 5% glycerol; 1 mM MgCl_2_, 0.1% Triton X-100) for 10 min at 65°C. Then, 15 µl of anti-2509 antibodies were added and the incubation was continued for 1 h on ice. The Dynabeads® Protein G beads (Invitrogen) were equilibrated in Co-IP buffer and added to the samples. After binding, the beads were washed three times with 1 ml of Co-IP buffer and boiled for 10 min in 50 µl of SDS-loading buffer. The proteins bound to the beads were separated on a 12% Tris/Tricine gel and analyzed using His-tag specific antibodies (Penta-His HRP Conjugate; Qiagen). The immunocomplexes were detected with the ECL kit (GE Healthcare).

### Determination of total protein synthesis

The strains PBL2025 and PBL2025Δ*2509* were grown in Brock`s medium to and OD_600_ of 1.5 and incubation was continued for 3 days in the same medium. Then, the cells were reinoculated in fresh Brock`s medium. After 3 h, [^35^S] methionine (20 µCi/ml) was added to 1 ml of the culture, and incubation was continued for 7 h at 65°C. The proteins were TCA precipitated and the incorporated radioactivity was measured in triplicate using a scintillation counter.

## RESULTS

### aIF2-γ bound to the 5′-P_3_-end of a leaderless mRNA impedes *in vitro* translation initiation complex formation

**Figure 1.**
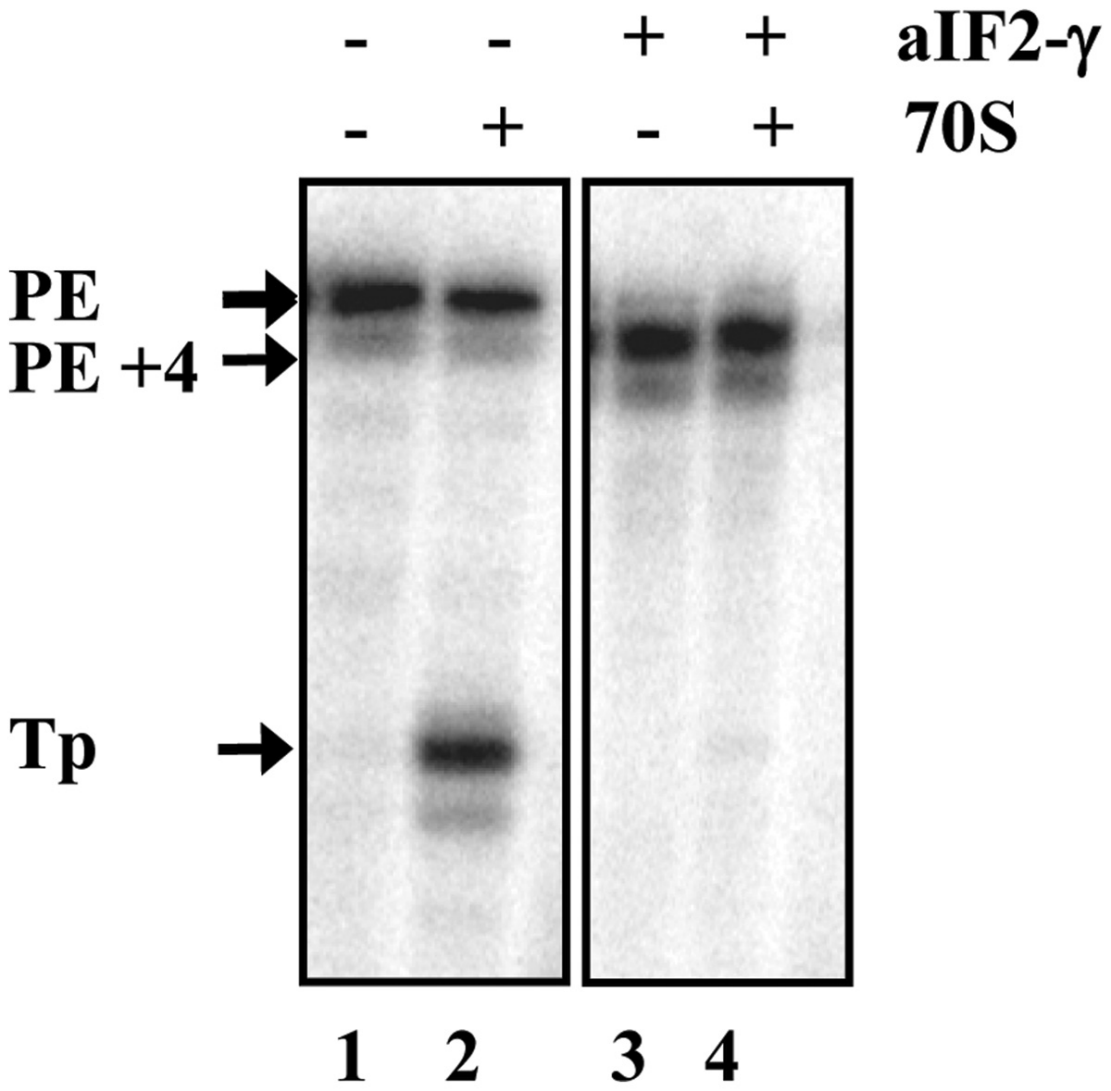
aIF2-γ inhibits *in vitro* translation initiation complex formation. Leaderless mRNA *104* was incubated without (lanes 1 and 2) and with (lane 3 and 4) aIF2-γ. Lanes 1 and 3, primer extension in the absence of 70S ribosomes. Lanes 2 and 4, toeprint analysis in the presence of 70S ribosomes and tRNA_f_^Met^. The primer extension signals obtained in the absence (PE) and presence of aIF2-γ (PE+4) as well as the toeprint signal (TP) obtained in the absence of aIF2-γ are marked by arrows.

According to our working model, aIF2/aIF2-γ serves to protect mRNAs from 5′ to 3′ directional decay under unfavorable conditions (15). As leaderless mRNAs are prevalent in Sso (3) and binding of aIF2/aIF2-γ to their 5′-end is anticipated to interfere with translation initiation, another assumption was that the release of aIF2/aIF2-γ from the 5′-end of leaderless mRNAs is particularly required for resumption of translation of this mRNA class. To test whether aIF2-γ interferes with translation initiation complex on leaderless mRNA, an *in vitro* toeprinting assay (18) was performed with the leaderless mRNA *104* (17). Efficient translation initiation complex formation on *E. coli* leaderless mRNAs was shown to commence with 70S monosomes (4). As purified Sso 70S ribosomes are unstable (20), *E. coli* 70S monosomes were used. As observed before (15), binding of the aIF2-γ subunit to the 5′-end of leaderless mRNA *104* resulted in an aIF2-γ specific stop signal of the reverse transcriptase at nucleotide position +4 (Figure 1, lane 3 and 4). Toeprinting performed in the absence of aIF2-γ revealed a ribosome-specific toeprint signal (Figure 1, lane 2), whereas no toeprint signal was obtained in the presence of aIF2-γ (Figure 1, lane 4), suggesting that 5′-end binding of the factor prevents the interaction of the start codon with the anti-codon of tRNAi. Although the toeprinting assay was performed at 37°C and with *E. coli* ribosomes, this observation prompted us to study the release of aIF2/aIF2-γ from the 5′-P_3_-end of RNA.

### Sso protein Sso2509 co-purifies with aIF2 during outgrowth from prolonged stationary phase

We considered that the release of aIF2/aIF2-γ from the 5′-P_3_-end of mRNA could either result from a posttranslational modification(s) or be caused by an auxiliary factor. According to our working model (15), aIF2/aIF2-γ is expected to be released from mRNAs during relief of nutrient stress, e.g. during outgrowth from prolonged stationary phase. Therefore, PH1-16(pMJ05- γ_His_) cells were grown to an OD_600_ of 1.5 (stationary phase) and incubation was continued for 3 days in the same medium (prolonged stationary phase) before His-tagged aIF2 was purified by means of Ni-affinity chromatography. In addition, the cells were harvested after prolonged stationary phase, reinoculated in fresh medium and further cultivated for 3 h (outgrowth) before purification of His-tagged aIF2. Mass spectrometry analysis of the aIF2-γ–specific bands obtained after gel-electrophoretic separation of the eluates obtained from either culture revealed only minor differences in posttranslational modifications (Supplementary Table S2).

**Figure 2.**
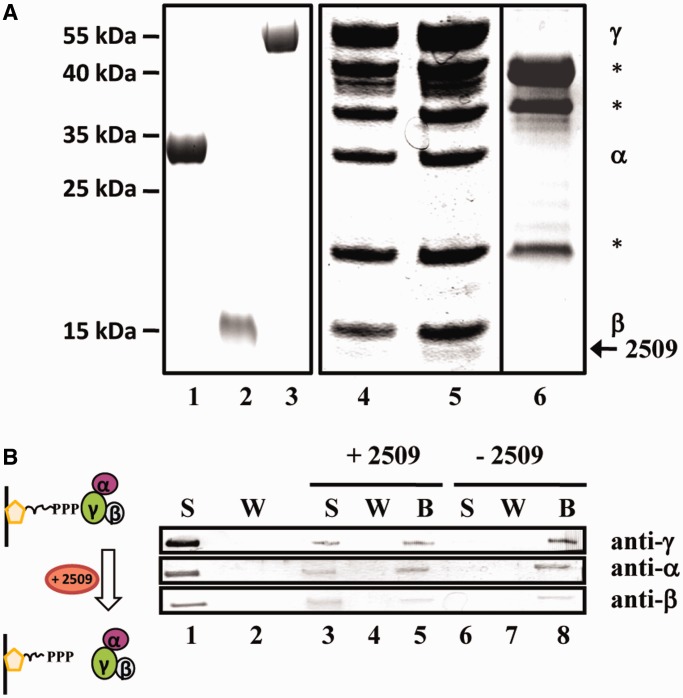
Interaction of Sso protein 2509 with aIF2. (**A**) Co-purification of Sso 2509 with aIF2 during outgrowth from prolonged starvation. Sso strain PH1-16(pMJ05-γ_His_) was subjected to prolonged stationary phase and then reinoculated in Brock’s medium containing arabinose as described in ‘Materials and Methods’ section. Purified aIF2-α (lane 1), aIF2-ß (lane 2) and aIF2-γ (lane 3) were separated on a 12% SDS-polyacrylamide gel. The trimeric factor containing aIF2-γ_His_ was captured by Ni-NTA chromatography from PH1-16(pMJ05-γ_His_) extracts obtained from cells subjected to prolonged starvation (lane 4) and 3 h after outgrowth from prolonged starvation (lane 5). The eluted proteins were separated on a 12% SDS polyacrylamide gel. A mock purification performed with extracts of strain PH1-16(pMJ05) obtained during outgrowth from prolonged stationary phase revealed nonspecific contaminant proteins (lane 6), which are indicated by stars. The bands corresponding to aIF2-α and aIF2-β co-purifying with aIF2-γ_His_ are indicated at the right. Protein Sso2509 (arrow) was found to associate predominantly with aIF2 during outgrowth. The positions of marker proteins are indicated at the left. The gels were stained with Coomassie brilliant blue. (**B**) Sso2509 removes aIF2 from the 5′-P_3_-end of RNA. The immobilized *2508*sh RNA was first saturated with aIF2. Unbound aIF2 present in the supernatant (S) was removed by several washing (W) steps. The presence and absence of aIF2 in the S (lane 1) and W (lane 2) fraction was confirmed by western blot analysis, respectively. The immobilized aIF2-2508sh complex was then either incubated with protein Sso2509 (lanes 3–5; + Sso2509) or with buffer (lanes 6–8; −Sso2509). The supernatant (S; lanes 3 and 6), the wash fraction (W; lanes 4 and 7) and the beads (B; lanes 5 and 8) were examined for the presence of aIF2(α,β,γ) by western blot analysis. The aIF2 subunits were detected using antibodies directed against either subunit protein.

With the aim to isolate a protein factor that co-purifies with aIF2, His-tagged aIF2 was purified from PH1-16(pMJ05- γ_His_) cultures as described above. In addition, a mock purification was performed from strain PH1-16(pMJ05) 3 h after outgrowth from prolonged stationary phase. When compared with the pattern of co-purifying proteins obtained from strain PH1-16(pMJ05- γ_His_) subjected to prolonged stationary phase and with the captured proteins from strain PH1-16(pMJ05) 3 h after outgrowth from prolonged stationary phase, an additional protein was detected in the aIF2 preparation obtained from PH1-16(pMJ05- γ_His_) cells 3 h after outgrowth from prolonged stationary phase (Figure 2A, lane 5). The protein was identified as Sso protein 2509. A BLAST search revealed that Sso 2509 is a member of the DUF35 family (**d**omain of **u**nknown **f**unction) and is conserved in Sulfobaceae (Supplementary Figure S1) as well as in other Archaea and Bacteria (not shown). As Sso2509 is annotated as a putative nucleic acid binding protein, we first tested whether it binds to RNA. However, using electrophoretic mobility shift assays and different RNA substrates, no RNA binding activity was observed (data not shown).

To verify whether expression of ORF *2509* is linked to growth/nutrient stress, the *2509* mRNA levels were determined in the Sso wild-type strain P2 during logarithmic growth (OD_600_ = 0.5), in stationary phase (OD_600_ = 1.5), and 3 h after outgrowth from prolonged stationary phase. When compared with logarithmic growth and stationary phase, the levels of *2509* mRNA were somewhat elevated during outgrowth from prolonged stationary phase (Supplementary Figure S2). This result could indicate that ORF *2509* expression increases during outgrowth. However, we did not further study the regulation of ORF *2509*.

### Release of aIF2 from the 5′-P_3_-end of RNA by Sso2509

Next, we tested whether purified Sso2509 can release aIF2 from the 5′-P_3_-end of RNA *in vitro*. 3′-biotinylated *2508*sh RNA containing a 5′-P_3_-end was attached to streptavidin-beads (Figure 2B) and the 5′-ends of the RNA were saturated with an excess of trimeric aIF2 (Figure 2B, lane 1). After removal of aIF2 from the supernatant (Figure 2B, lane 2), either Sso2509 or buffer was added to the streptavidin-RNA-aIF2 complex. Then, the supernatant, the wash fraction and the beads (retained fraction) were tested for the presence of the three aIF2 subunits using western blot analysis. Either subunit (∼50%) was released from the streptavidin-RNA-aIF2 complex into the supernatant on addition of protein Sso2509 (Figure 2B, lane 3), whereas in the control experiment, aIF2 remained bound to the beads (Figure 2B, lane 8).

To verify the release of aIF2 from RNA by Sso2509, acidic native polyacrylamide gel electrophoresis was used (7). The individual subunits of aIF2 are positively charged under acidic conditions and the formation of the trimeric complex results in migration toward the anode, relative to the individual subunits (Supplementary Figure S3, lanes 1–4). The presence of Sso2509 did not affect the migration properties of trimeric aIF2 complex under these conditions. On addition of total RNA to aIF2, the RNA-aIF2 complexes did not enter the gel, which was most likely caused by the increase in negative charge (Supplementary Figure S3, lane 7). However, incubation of the RNA-aIF2 complexes with protein Sso2509 resulted in apparent release of aIF2 from RNA as indicated by a reentry of aIF2 into the gel (Supplementary Figure S3, lane 8).

Sso aIF2 was shown to protect mRNA from 5′ to 3′ directional decay by the exoribonuclease aCPSF2 (16). We therefore also asked whether Sso2509 attenuates aIF2-mediated stabilization. The 5′ to 3′ exonucleolytic decay by recombinant aCPSF2 of a 5′ terminally labeled 42-nt-long synthetic RNA (5′-PPP-GGA*-‘-3’) resulted in a single nucleotide as final degradation product (Supplementary Figure S4, lanes 7–11). The addition of Sso2509 to the reaction mix did not alter the velocity of aCPSF2 in degrading the substrate (Supplementary Figure S4, lanes 12–16). As shown before (16), the aIF2-RNA complex attenuated 5′ to 3′ decay, which was discernable ∼15 min after addition of the enzyme (Supplementary Figure S4, lanes 17–21). However, when Sso2509 was added to the aIF2-RNA complex before addition of aCPSF2, degradation was noticeable already 5 min after addition of the enzyme, i.e. aIF2-mediated protection was abrogated (Supplementary Figure S4, lanes 22–26). Taken together these studies indicated that Sso2509 stimulates the release of aIF2 from the 5′-P_3_-end of RNAs.

### Sso2509 and aIF2-γ interact directly

**Figure 3.**
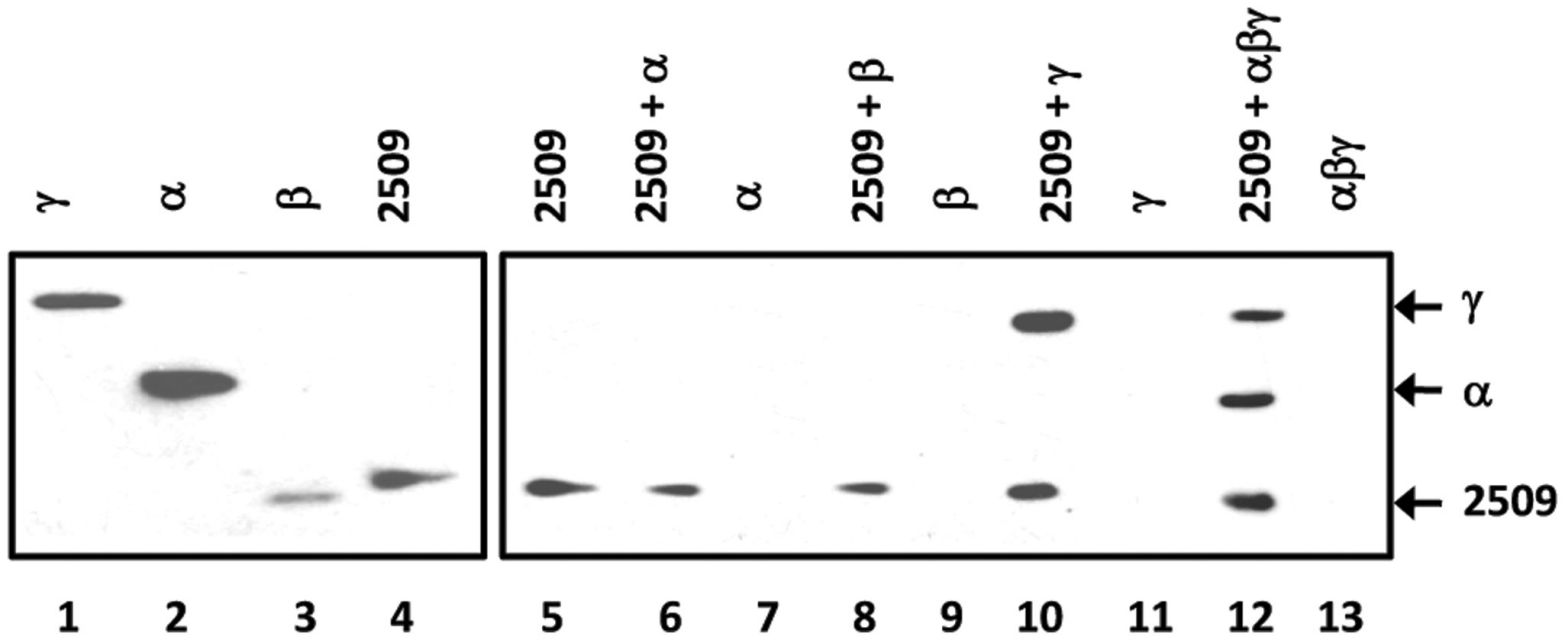
Interaction of Sso2509 with aIF2-γ. Lane 1–4, 2.5 pmol of either purified aIF2 subunit and Sso2509 (1/40 of input) were loaded on a 12% polyacrylamide gel followed by western blot analysis with anti-His antibodies. Lane 5, Immunoprecipitation of Sso2509 with anti-2509 antibodies and detection by western blot analysis with anti-His antibodies. Lanes 6, 8, 10 and 12, Co-IP experiments using anti-2509–specific antibodies. Sso 2509 was incubated together with aIF2-α (lane 6), aIF2ß (lane 8), aIF2-γ (lane 10) and trimeric aIF2 (lane 12), respectively. The eluates were separated on a 12% SDS polyacrylamide gel and the proteins were detected by western blot analysis using anti-His antibodies. Lanes 7, 9, 11, 13, mock experiments in the absence of protein Sso2509. The positions of the aIF2-γ, aIF2-α and 2509-specific bands are indicated by arrows.

Next, we tested whether a direct interaction of Sso2509 with aIF2-γ could explain the observed release of aIF2 from RNA. To address this experimentally, a Sso2509-specific antibody was raised, which was used in Co-IP studies. Sso2509 was added to the individual aIF2 subunits as well as to reconstituted trimeric aIF2, whereas protein Sso2509 was omitted in the control experiments (Figure 3, lanes 7, 9, 11 and 13). Then, anti-2509 antibodies were added, which was followed by addition of protein G beads. A magnetic device was used to pull down the immunocomplexes, which were then analyzed for proteins that co-precipitated with Sso2509. The immunodetection was performed with anti-His antibodies, as all proteins contained a His-tag. The sole aIF2-α and aIF2-ß subunits (Figure 3, lanes 6 and 8) did not interact with Sso2509, whereas aIF2-γ co-immunoprecipitated with Sso2509 (Figure 3, lane 10). Similarly, the aIF2-α and aIF2-γ subunits were co-immunoprecipitated (Figure 3, lane 12) when trimeric aIF2 was used in the assay. Interestingly, the aIF2-β subunit was absent in the co-immunoprecipitate when trimeric aIF2 was used (Figure 3, lane 12). Taken together, these experiments revealed a direct interaction between Sso2509 and the aIF2-γ subunit.

### Impaired protein synthesis during outgrowth of a Sso *2509* deletion mutant

**Figure 4.**
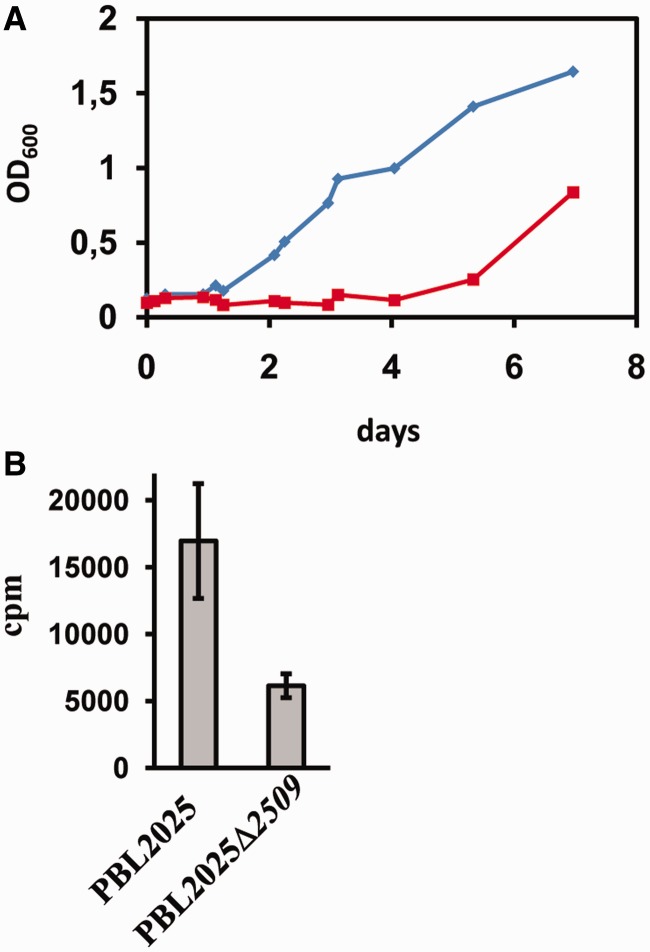
Impaired outgrowth of and total protein synthesis in a Sso *2509* deletion mutant. (**A**) Strains PBL2025 and PBL2025Δ*2509* were grown as described in the text. Outgrowth of the wild-type strain (diamonds) and the *2509* deletion mutant (squares) was monitored after the cells were subjected to prolonged stationary phase. (**B**) Protein synthesis during outgrowth is impaired in the *2509* deletion strain. The error bars represent standard deviations from three independent experiments.

To address the physiological relevance of Sso2509, ORF *2509* was deleted in Sso strain PBL2025 by gene replacement (21). Strain PBL2025Δ*2509* and strain PBL2025 were inoculated in Brock’s medium. The growth of both strains was indistinguishable in exponential growth phase (Supplementary Figure S5). In contrast, when both strains were subjected to prolonged stationary phase and outgrowth was monitored after reinoculation in fresh medium, outgrowth of strain PBL2025Δ*2509* was considerably delayed (Figure 4A). In addition, when compared with the parental strain, strain PBL2025Δ*2509* showed a reduced *de novo* synthesis of proteins after reinoculation in fresh medium as monitored by overall incorporation of [^35^S] methionine (Figure 4B). Thus, the delayed outgrowth of strain PBL2025Δ*2509* likely results from an impaired protein synthesis. As Sso2509 is apparently required for fast(er) resumption of translation after recovery from nutrient stress, the protein was termed Trf.

## DISCUSSION

The Trf identified in this study contains an N-terminal rubredoxin-like Zn-binding domain and a C-terminal oligonucleotide binding (OB)-fold domain. The DUF35 protein family is highly conserved in *Sulfolobacaea* (Supplementary Figure S1) and multiple orthologous and paralogous proteins are found in different organisms (string-db.org; COG1545). Although six paralogues genes are present in Sso, only Sso2509 was co-purifying with aIF2. Nevertheless, it could be interesting to ask whether 5′-end protection by aIF2/aIF2-γ, the presence of Trf and the abundance of leaderless mRNAs are interlinked in different Archaea.

Proteins containing OB-fold domains and/or rubredoxin-like Zn-binding domains are frequently found in proteins involved in translation. For instance, a zinc binding domain is present in eIF2-γ and eIF2-β (8). We noticed that only the aIF2-α and aIF2-γ subunits co-immunoprecipitated with Trf but not the β-subunit (Figure 3, lane 12). Taken with the absence of contacts between aIF2-α and aIF2-β (22), this observation could suggest that Trf binding to aIF2-γ displaces aIF2-β. The C-terminal part of Sso aIF2-β is highly flexible and contacts with its Zn-binding domain the aIF2-γ G-domain (22). Thus, it seems worth testing whether the Zn-binding domain of Trf is required for the interaction with aIF2-γ.

In contrast to favorable chemoorganotrophic growth, Sso can also thrive in sulfur-rich hot springs, wherein it grows chemolithotrophically with a slow rate. We have previously speculated that free aIF2/aIF2-γ (not engaged in translation) counteracts 5′ to 3′ mRNA decay (15) under conditions where translation ceases, i.e. in late stationary phase or during stringent control (23). This hypothesis was supported by the identification of Sso aCPSF2, degrading mRNA with 5′ to 3′ directionality (16). Using quantitative western blotting we did not observe an increase of aIF2-γ in late stationary phase when compared with logarithmically growing cells (B. Märtens, unpublished). However, this observation is not necessarily conflicting with our working hypothesis, as the relocation of aIF2/aIF2-γ from the translational apparatus to the 5′-end of mRNAs might be sufficient to protect from 5′ to 3′ decay.

5′-end protection of mRNAs by aIF2 during growth/nutrient stress or slow chemolithotrophic growth seems to provide one means to protect the integrity of mRNAs. In addition, in the presence of NDPs the Sso exosome adds A-rich tails at the 3′-end of mRNAs (24). Thus, at reduced ATP levels this mode of action might slow down 3′ to 5′ degradation by the exosome, and contribute as well to mRNA stabilization.

**Figure 5.**
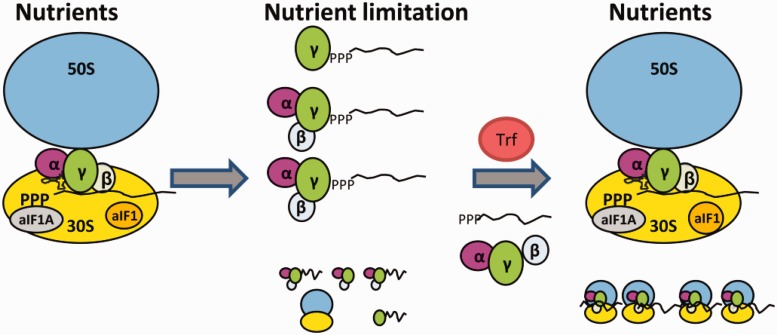
Model for RNA 5′-end protection by aIF2 and its removal by Trf. During optimal growth conditions (Nutrients), aIF2 binds predominantly to the ribosome, which is assisted by aIF1/aIF1A (9). Ribosome bound aIF2 then recruits tRNAi to the translation initiation complex. During nutrient limitation, overall protein synthesis ceases, which in turn results in increased levels of free aIF2. 5′-end protection of mRNAs by either trimeric aIF2 or by aIF2-γ might be of particular importance to maintain the integrity of leaderless mRNAs. On relief of nutrient stress, Trf binds to aIF2-γ, and aIF2/aIF2-γ is released from the 5′-P3-end of mRNAs. The aIF2-free mRNAs can then bind to ribosomes and translation resumes.

The protection of mRNAs has also been described in Eukaryotes as a means to permit fast adaptation to environmental changes. During impaired translation or in stationary phase, mRNAs can enter P-bodies. In addition to their function as dedicated foci for mRNA de-capping and degradation, they also serve to store mRNAs. These mRNAs can reenter translation after stress recovery (25). The release of aIF2 by Trf during recovery from nutrient stress could particularly allow anew translation of leaderless Sso mRNAs during adaptation to fast(er) growth (Figure 5). The observations that outgrowth and protein synthesis were reduced in a Sso *trf* (*2509*) deletion mutant after shift to fresh medium would be in line with this notion.

## SUPPLEMENTARY DATA

Supplementary Data are available at NAR Online.

## FUNDING

Austrian Science Fund (FWF) [P21560 to U.B.]. Funding for open access charge: FWF.

*Conflict of interest statement*. None declared.

## Supplementary Material

Supplementary Data
