# Supplementary material for: Back to translation: removal of aIF2 from the 5′-end of mRNAs by translation recovery factor in the crenarchaeon Sulfolobus solfataricus
Source: Nucleic Acids Res. 2013 Nov 23;42(4):2505–11. doi: 10.1093/nar/gkt1169 (PMC3936769; doi:10.1093/nar/gkt1169)
Supplement: Supplementary Data [file supp_42_4_2505__index.html]

Back to translation: removal of aIF2 from the 5′-end of mRNAs by translation recovery factor in the crenarchaeon Sulfolobus solfataricus — Back to translation: removal of aIF2 from the 5′-end of mRNAs by translation recovery factor in the crenarchaeon Sulfolobus solfataricus — Supplementary Data 

# Back to translation: removal of aIF2 from the 5′-end of mRNAs by translation recovery factor in the crenarchaeon *Sulfolobus solfataricus*

## Supplementary Data

files

**Files in this Data Supplement:**

- Supplementary Data - pdf file
