# Supplementary material for: Back to translation: removal of aIF2 from the 5′-end of mRNAs by translation recovery factor in the crenarchaeon Sulfolobus solfataricus
Source: Nucleic Acids Res. 2013 Nov 23;42(4):2505–11. doi: 10.1093/nar/gkt1169 (PMC3936769; doi:10.1093/nar/gkt1169)
Supplement: Supplementary Data [file supp_gkt1169_nar-01826-v-2013-File007.pdf]

Märtens *et. al*

Supporting Information

Construction of strain PBL2025Δ*2509*

The Sso 2509 deletion strain was constructed as follows. The up- (1172 bp) and downstream (2079 bp) flanking regions of *Sso 2509* were amplified by PCR from genomic DNA isolated from Sso PBL2025 (17). The upstream flanking region was amplified with oligonucleotides 5´-TCTGGTACCGAACAAGGTTTTTTAATAGGTAGTA-3´, (contains a KpnI restriction site) and 5´-TCTCCATGGCGCTCACCCAATACTAAAGTTACAC-3´ (contains a NcoI restriction site). The PCR product was cleaved with KpnI and NcoI and ligated into the corresponding sites of plasmid pET2268 (17), containing the *lacS* cassette with its authentic promoter- and terminator region. This yielded plasmid pET2268-2509up. The downstream flanking region was amplified with oligonucleotides 5´-TCTGGATCCCTTTTAATCATTTCACAATATGATC-3´ (contains a BamHI restriction site) and 5´-TCTGCGGCCGCACCTTTATTCTGTACTCTTCTAAT-3´ (contains a NotI restriction site). The PCR product was cleaved with BamHI and NotI and ligated into plasmid pET2268-2509up, yielding plasmid pET2268-Δ*2509.* The plasmid was transformed into Sso PBL2025 as described (17). After electroporation, the cells were resuspended in 1 ml of demineralized water, incubated briefly on ice, and then incubated 10 min at 75°C. The cells were transferred to 50 ml pre-warmed lactose minimal medium (Brock´s salts, supplemented with 0.4 % lactose). After the culture reached an OD_{600} of 0.1, 3 ml of the culture were transferred to 50 ml of fresh lactose minimal medium. After the cells reached an OD_{600} of ~ 0.4, the cells were plated and single blue colonies were inoculated in Brock´s medium. After the first selection a mixture of the wild-type gene and the knockout allele was obtained as described (17). The plating/inoculation of single colonies in selective medium was repeated 3 times to single out the deletion strain. The deletion strain PBL2025Δ*2509* was confirmed by sequencing the PCR product (2242 bp) obtained with oligonucleotides 5´-AGGTAGTTTAAATACTGGTCAGCCC-3´ and 5´-ATAGCCCGTCATCAGTATGGAGTTC-3´.

Construction of plasmid pMJ05-γ_{His} and transformation of Sso cells

The aIF2-γ gene (Sso *0412*) was amplified by PCR using genomic DNA of Sso strain P2 as template together with the oligonucleotides 5´-AGACCATGGCATGGCCTAAAGTTCAACCAG -3´ (contains a Nco1 restriction site) and 5´-AAAGGATCCGATCTCTACTAAACCCCATC-3´ (contains a BamH1 restriction site). The PCR product was cleaved with NcoI and BamHI, and ligated into the corresponding sites of plasmid pSVA5 (18), resulting in plasmid pSVA5-γ_{His}. Plasmid pSVA5-γ_{His} was cleaved with Eco52I and XmaJI, and the resulting fragment was ligated into the corresponding sites of plasmid pMJ05 (18), which gave rise to plasmid pMJ05-γ_{His}. The coding sequence of the aIF2-γ gene is preceded by an arabinose-inducible promotor (18). 100 ng of the respective plasmid DNA was used for electroporation of Sso PH1-16 (Δ*pyrEF*; Δ*lacS*) cells as described (18). The transformants were selected in medium without uracil. After reaching an OD_{600} of 0.5, the cells were plated and single colonies were inoculated in liquid media without uracil. Genomic DNA was isolated and the presence of the intact plasmid was confirmed using the pMJ05 specific primers 5´-GGATGCTAAACAACTATTCAAACTG-3´ and 5´-GTTGTGTGGAATTGTGAGCGGATAA-3´.

Sso-aCPSF2 degradation assay.

The 42-nt-long RNA (5‘- PPP-GGA* -“-3) harboring a single radioactive labeled A nucleotide at position +3 at the 5´end was synthesized as follows: The oligonucleotide (5’-GGGCTCTAGAGTAATACGACTCACTATAGG-3’), containing a T7-promoter was hybridized to the oligonucleotide (5’-AGACAGAAACCACAGAACGAGACAGAAACCACAGAACGTCCTATAGTGAGT CGTATTAC-3’). The duplex was used as template for *in vitro* transcription using the Ambion MEGAshortscript T7 Kit together with [α-^{32}P]ATP. The RNA was then loaded onto a 12 % polyacrylamide gel containing 8 M urea and purified following a standard

protocol. The RNA degradation assays were carried out as recently described (16). Briefly, aCPSF2-activity was assayed in a 5 µl reaction volume by incubating the RNA (0.05µM/0.25 µM) with 100 ng of enzyme for 0 to 60 min at 65° C in reaction buffer (5 mM HEPES pH 7, 10 mM KCl, 10 mM $Mg[OAc]_2$). The aIF2 (0.5 µM) alone or together with 2509 protein (2.5 µM) were added to the reaction mixture and incubated for 2 min at 65°C before addition of aCPSF2. All proteins were pre-incubated 10 min at 65°C prior to the addition of RNA. The reactions were terminated by addition of 5 µl of 95 % formamide/20 mM EDTA and analyzed on 20 % PAA–7 M urea gels.

Native acidic PAGE

The gel was prepared as described [9]. 30 pmol of α-, β- and γ-proteins alone or together with 2509 or 0.5 µg total RNA were incubated in buffer B (50 mM Tris–HCl, pH 6.0, 30 mM KCl, 0.5 mM $MgCl_2$). The proteins and the complexes were visualized by non-denaturing electrophoresis on 12 % polyacrylamide gels prepared in acetate buffer (120 mM potassium acetate and 72 mM acetic acid, pH 4.3). The gels included a stacking overlay of 4 % polyacrylamide in acetate buffer (120 mM potassium acetate and 12 mM acetic acid, pH 6.8). As running buffer 133 mM acetic acid and 350 mM β-alanine, pH 4.4 was used. After the run, the gels were stained with Coomassie brilliant blue.

RT-PCR

Total RNA from Sso strain P2 was isolated using Trizol (Invitrogen) and DNaseI (Roche) treated. The cDNA was synthesized using 1 µg total RNA together with random hexonucleotides (Thermo scientfic) and SuperScriptIII reverse transcriptase (Invitrogen) according to the manufacturer’s instructions. The PCR reaction with GoTaq® Green Master Mix (Promega) was performed with the *2509*-specific oligonucleotides 5´-ATGAGGGAAGAAGAAATAAGAG-3´ and 5‘-CCTAACTTCAATATCTGAAGAACC-3´. Sso *0764* (encodes Adh2) was amplified with the oligonucleotides (5´-CTCCTATCACTTTCGCGCCAAG-3´ and 5´-TTGGCACTAACGTTCCAGACG-3´) and served as internal control.

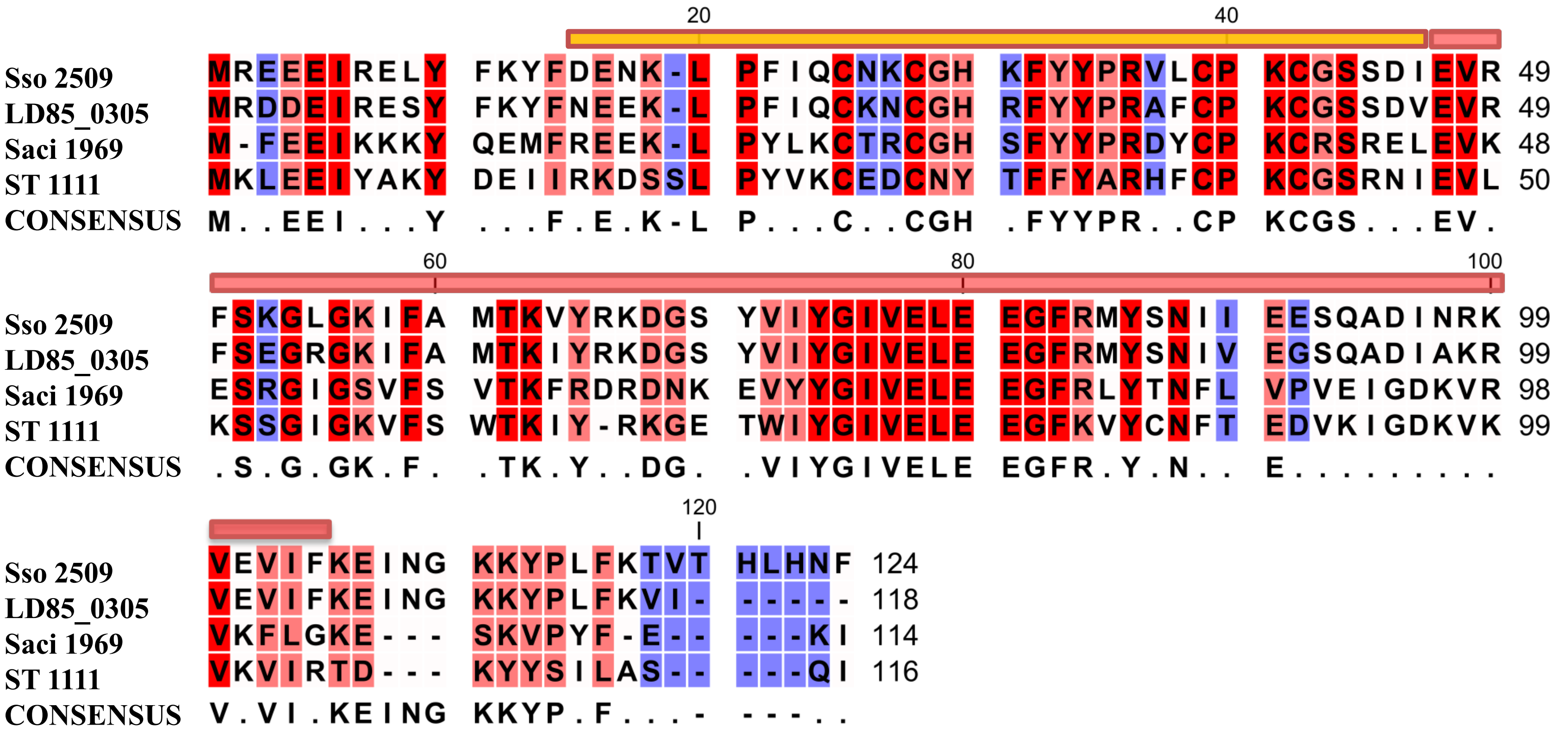

Supplementary Figure S1. Domains and conservation of Sso2509 in Sulfolobaceae. Alignment of Sso2509 orthologs from *Sulfolobus islandicus* (LD85_0305), *Sulfolobus acidocaldarius* (Saci 1969) and *Sulfolobus tokadaii* (ST 1111) using CLC viewer software. The rubredoxin-like domain (amino acids 15-47) and the OB-fold domain (amino acids 47-105) are shown by an orange and a red bar, respectively.

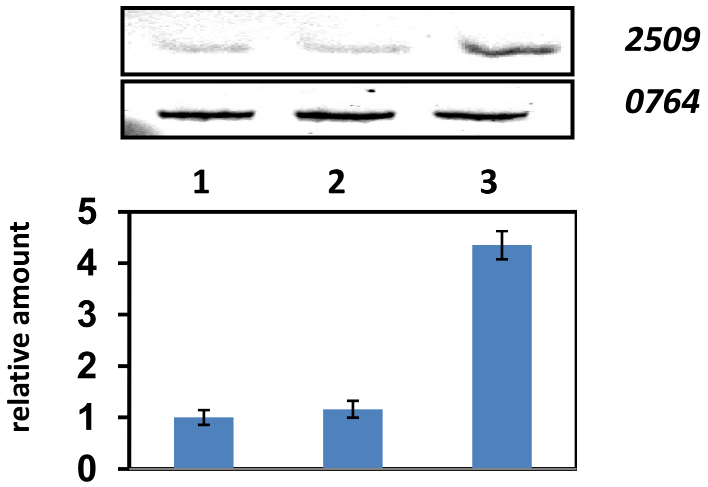

Supplementary Figure S2. Abundance of *2509* mRNA during logarithmic growth (OD_{600} = 0.5; lane 1), in stationary phase (OD_{600} = 1.5; lane 2) and 3 h after outgrowth from prolonged stationary phase (lane 3). Upper panel: representative RT-PCR experiment. Lower panel: Quantification of the results from triplicate experiments. The level of *2509* mRNA obtained during logarithmic growth was set to 1. The error bars represent standard deviations. ORF *0764* (Adh2) was amplified and used as ínternal standard.

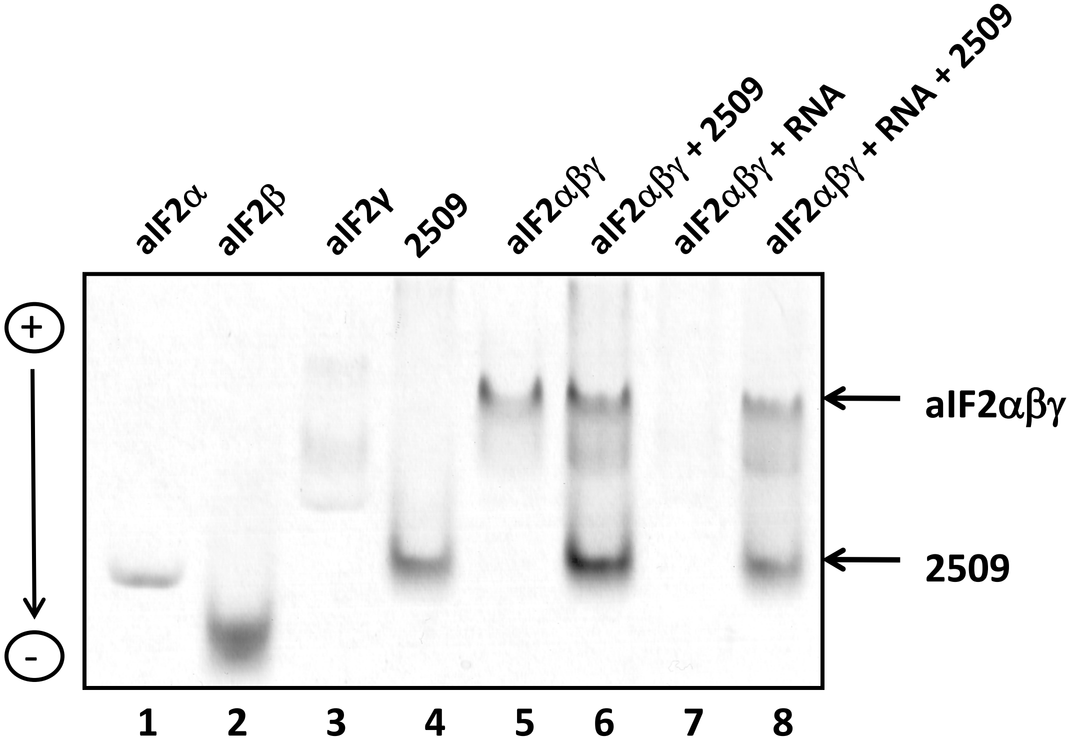

Supplementary Figure S3. Protein 2509 releases aIF2 bound to RNA. Acidic native gel to test the release by protein Sso2509 of aIF2 bound to RNA. Lanes, 1-3, separation of either aIF2 subunit. Lane 4, input concentration of protein Sso2509 used in the experiment. Lane 5, formation of the trimeric complex (aIF2αβγ) upon incubation of the individual subunits (aIF2α, aIF2β and aIF2γ) at 65°C for 10 min. Lane 6, the addition of protein Sso2509 did not change the mobility of trimeric aIF2. Lane 7, upon incubation of aIF2 with RNA, the RNA-aIF2 complex did not enter the gel. Lane 8, upon addition of protein Sso2509 aIF2 was apparently released from RNA and re-entered the gel (lane 8).

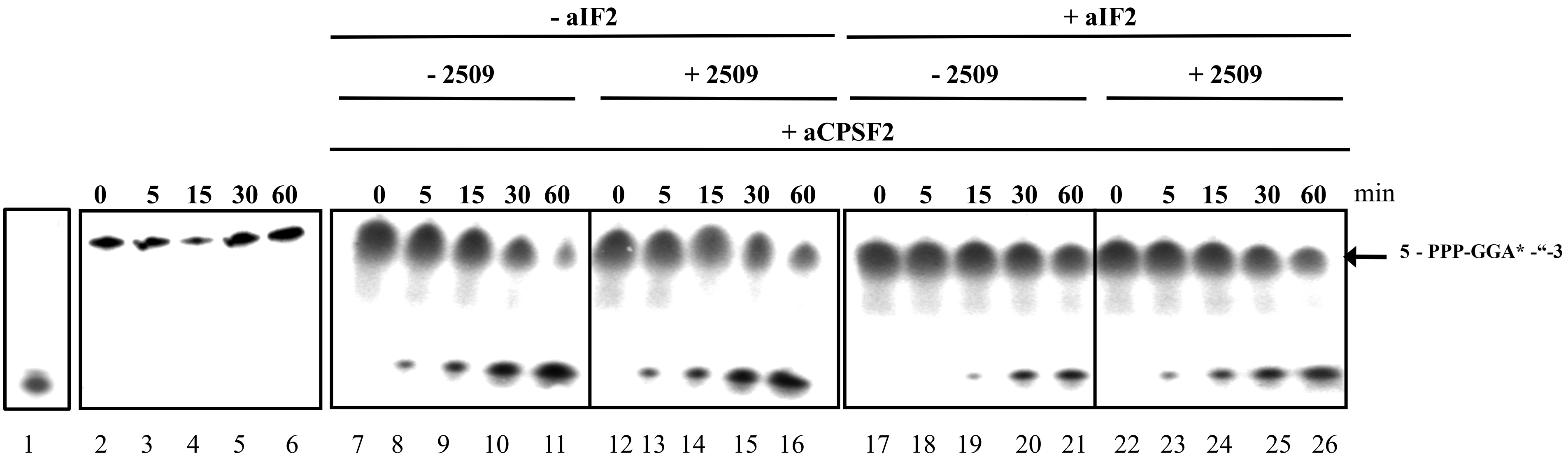

Supplementary Figure S4. Protein Sso2509 abrogates 5 end protection of RNA conferred by aIF2 against the exoribonuclease aCPSF2. The 42-nt-long RNA (5 - PPP-GGA* -“-3) harboring a single radioactive labeled A nucleotide at position +3 at the 5 end was *in vitro* transcribed and gel-purified. The radiolabeled RNA 5 - PPP-GGA* -“-3 (0.05 µM in lane 2-6 or 0.25 µM in lane 7-26) was incubated for 0-60 min at 65 C in reaction buffer (lanes 2-6), in the presence of 4 µM aCPSF2 (lane 7 - 26) or in the presence of aCPSF2 (4 µM) and 2.5 µM Sso2509 (lanes 12-16). Lanes 17-21, the aIF2 trimer (0.5 µM) was incubated with RNA (0.25 µM) in the absence of protein Sso2509. Lanes 22-26, the aIF2 trimer (0.5 µM) was incubated with RNA (0.25 µM) in the presence of protein Sso2509 (2.5 µM). Lanes 17-26, after incubation of the components for 2 min at 65 C aCPSF2 (4 µM) was added. The reactions were terminated through boiling in loading buffer and loaded together with [^{32}P]ATP (lane 1) on a 20 % PAA-7 M urea gel.

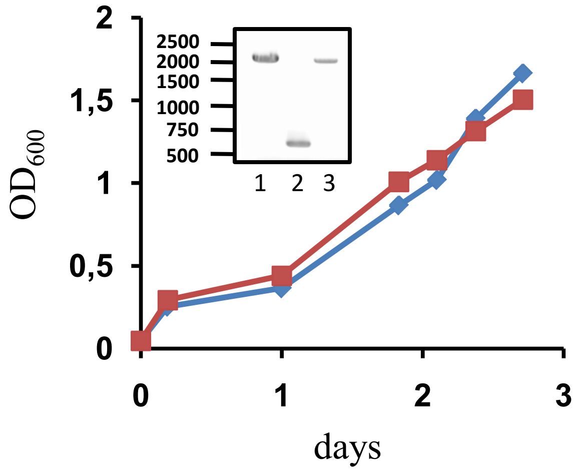

Supplementary Figure S5. Growth of strains PBL2025 (diamonds) and PBL2025Δ*2509* (squares) during exponential growth. Inset: Deletion of ORF *2509* in strain PBL2025Δ*2509* The PCR was performed with plasmid pET2268-Δ*2509* (lane1), with genomic DNA isolated from strain PBL2025 (lane 2) and with genomic DNA isolated from strain PBL2025Δ*2509* (lane 3) as described in MATERIALS and METHODS. The PCR product was sequenced to confirm the deletion in strain PBL2025Δ*2509*.
